# Global Longitudinal Strain in Type 1 and Type 2 Diabetes: Why Similar Abnormalities May Not Mean the Same Thing

**DOI:** 10.1111/echo.70657

**Published:** 2026-10-28

**Authors:** Zuhal Fazli, Vivetha Pooranachandran

**Affiliations:** ^1^ The Royal Wolverhampton Hospital Wolverhampton UK; ^2^ Department of Natural Sciences Middlesex University London UK; ^3^ Sheffield Teaching Hospital Sheffield UK

**Keywords:** diabetic cardiomyopathy, global longitudinal strain, speckle‐tracking echocardiography, type 1 diabetes, type 2 diabetes

## Abstract

Global longitudinal strain (GLS) is increasingly used to detect subclinical myocardial dysfunction in diabetes, but similar abnormalities may not carry the same biological meaning in type 1 diabetes (T1D) and type 2 diabetes (T2D). In T1D, chronic hyperglycemia, oxidative stress, microvascular dysfunction, and progressive interstitial remodeling may contribute prominently to impaired myocardial deformation. In T2D, insulin resistance, adiposity, lipotoxicity, mitochondrial dysfunction, and altered myocardial energetics may have greater influence, although substantial mechanistic overlap exists. This review examines how these differing pathophysiological contexts influence the interpretation of GLS, the evolution of myocardial dysfunction, and reported responses to contemporary cardiometabolic therapies. Complementary deformation parameters are also considered where they provide additional insight into ventricular mechanics. Importantly, GLS is a sensitive marker of myocardial dysfunction but is not mechanism‐specific and may be influenced by metabolic, hemodynamic, and loading conditions. Interpretation should therefore incorporate diabetes subtype, disease duration, glycemic burden, comorbidity, and complementary imaging findings. A mechanistically informed approach may improve the clinical and research interpretation of myocardial deformation in diabetes.

## Graphical abstract

In T1D, chronic hyperglycaemia, oxidative stress, microvascular dysfunction, and interstitial remodelling predominate. In T2D, insulin resistance, adiposity, lipotoxicity, altered substrate utilisation, and impaired energetics are more prominent. GLS is therefore a sensitive, but not mechanism‐specific, marker of early myocardial dysfunction.

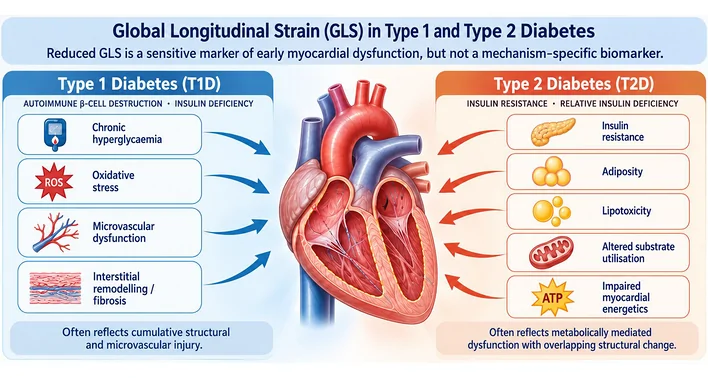

**Abbreviations**
AGE–RAGE: advanced glycation end product–receptor for advanced glycation end products
EAT: epicardial adipose tissue
GCS: global circumferential strain
GLS: global longitudinal strain
GRS: global radial strain
LSS: layer‐specific strain
LVEF: left ventricular ejection fraction
PSD: peak strain dispersion
SGLT2: sodium‐glucose cotransporter 2
T1D: type 1 diabetes
T2D: type 2 diabetes

## 1 Introduction

Global longitudinal strain (GLS) is increasingly used to identify subclinical myocardial dysfunction in diabetes, but an abnormal GLS value does not necessarily represent the same myocardial process in type 1 diabetes (T1D) and type 2 diabetes (T2D) [1, 2]. Understanding the underlying disease biology is therefore important when interpreting deformation findings.

Myocardial deformation imaging evaluates how different layers of the left ventricle shorten, thicken, and coordinate during the cardiac cycle. Among these measures, GLS is particularly sensitive to early systolic dysfunction because it predominantly reflects longitudinal myocardial shortening, which may be affected before conventional measures such as left ventricular ejection fraction (LVEF) [3]. A less negative GLS value indicates impaired fiber shortening even when LVEF remains normal [4].

Other deformation parameters provide complementary information. Global circumferential strain (GCS) reflects mid‐wall fiber shortening, while global radial strain (GRS) reflects wall thickening (Figure 1) [5]. These layers often compensate when longitudinal fibers fail, which is why LVEF can remain normal despite significant subendocardial injury [6]. Timing‐based indices such as peak strain dispersion, assess how synchronously different segments contract, and layer‐specific strain separates endocardial, mid‐myocardial, and epicardial deformation to reveal early transmural involvement [7, 8].

**FIGURE 1:**
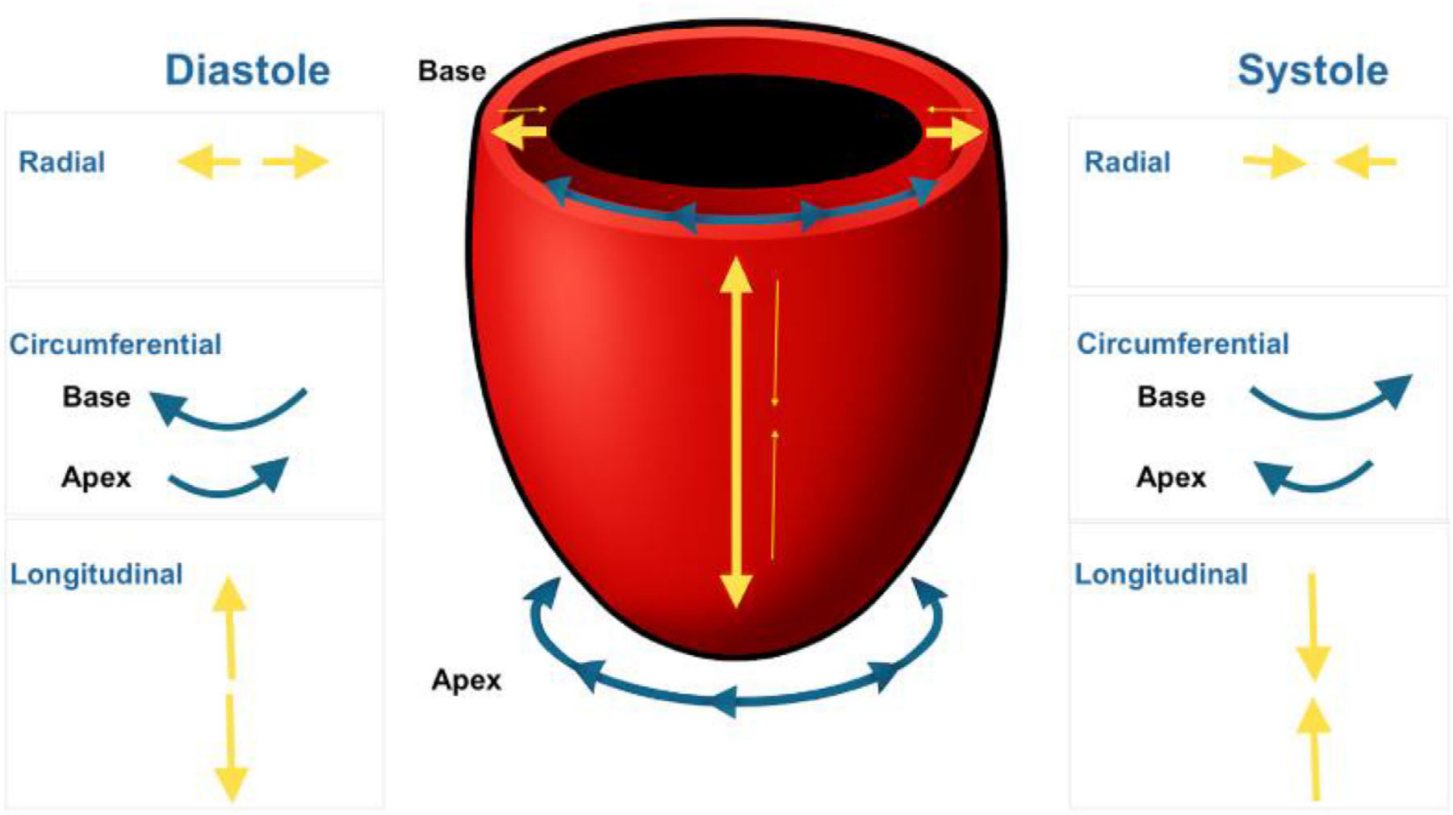
Principal components of left ventricular myocardial deformation. Longitudinal strain represents shortening of the myocardium along the long axis, circumferential strain reflects shortening around the ventricular circumference, and radial strain represents myocardial wall thickening. During systole, longitudinal and circumferential strain become more negative as the myocardium shortens, whereas radial strain becomes positive as the ventricular wall thickens. These complementary deformation components describe different aspects of left ventricular mechanics and may be affected differently during the development of diabetic myocardial dysfunction.

These parameters are not new, but their interpretation may differ when T1D and T2D are considered separately, because the relative contribution of metabolic, microvascular and structural abnormalities differs between the two conditions. Each subtype stresses the myocardium through distinct biological pathways, and these pathways map directly onto the deformation patterns described in the following sections [9]. In this way, strain imaging provides not only an early functional marker but also a pathophysiological lens, revealing how metabolic, microvascular, and structural stresses differ between T1D and T2D. Understanding what each parameter measures is therefore essential, because diabetes does not affect all myocardial layers equally, and recognizing these subtype‐specific patterns prevents misclassification and missed opportunities for early intervention.

Rather than reviewing whether GLS is impaired in diabetes, this review examines whether similar abnormalities in myocardial deformation carry the same biological significance in T1D and T2D. By integrating deformation imaging with current understanding of diabetic myocardial biology, we propose a framework for interpreting GLS within the context of diabetes subtype.

## 2 Mechanistic Substrate of Myocardial Injury in Type 1 Diabetes

Although T1D and T2D share several pathways of myocardial injury, the relative contribution of individual mechanisms differs between the two conditions [10]. In T1D, chronic hyperglycemia, oxidative stress, microvascular dysfunction and progressive interstitial remodeling appear to contribute prominently to myocardial injury [11]. These processes may begin early in the disease course and produce subtle myocardial dysfunction, with longitudinal deformation frequently affected despite preserved conventional systolic function [12].

Persistent hyperglycemia is central to myocardial injury in T1D. Absolute insulin deficiency exposes the myocardium to prolonged glucotoxicity, activating the polyol pathway, protein kinase C, and AGE–RAGE signaling [13]. These pathways increase oxidative stress and promote collagen cross‐linking within the interstitium [14]. These pathways may promote progressive interstitial fibrosis and reduced myocardial compliance, linking chronic metabolic exposure to subsequent structural remodeling [13]. This gradual accumulation of interstitial fibrosis stiffens the myocardium and reduces contractile efficiency, but because the injury is diffuse and slowly progressive, conventional measures such as ejection fraction remain normal for many years [15].

Microvascular dysfunction is another defining feature. Microvascular structural abnormalities, including capillary basement membrane thickening, have been demonstrated in peripheral tissues in diabetes, including skin and skeletal muscle, supporting the presence of systemic microangiopathic remodeling in longstanding T1D [16, 17]. These changes preferentially affect the subendocardium, where perfusion reserve is lowest and metabolic demand is highest [18]. The result is chronic, low‐grade subendocardial ischemia that does not cause infarction but does impair longitudinal fiber shortening [18]. This mechanism aligns closely with the imaging phenotype: longitudinal strain falls early, while circumferential and radial mechanics remain preserved because mid‐wall fibers are less dependent on microvascular reserve [13].

## 3 Myocardial Deformation Pattern in Type 1 Diabetes

Across pediatric and adult cohorts, early impairment of GLS and abnormalities in diastolic function are frequently reported in T1D, although the pattern of myocardial deformation is heterogeneous and circumferential, radial and rotational abnormalities have also been described. This pattern appears across imaging modalities and age groups, reflecting a biology shaped by chronic hyperglycemia, microvascular dysfunction, oxidative stress, and progressive interstitial remodeling [13].

The earliest evidence comes from childhood, where deformation abnormalities are already detectable despite normal ejection fraction and unremarkable conventional imaging. In a cohort of 100 children with T1D, GLS was reduced while circumferential and radial strain remained preserved, and the degree of impairment tracked closely with HbA1c rather than disease duration [19]. A meta‐analysis of 755 pediatric patients confirmed this pattern, reporting a pooled GLS reduction of around three percentage points and identifying glycemic burden as the dominant determinant [20]. These findings place the onset of T1D‐related myocardial dysfunction early in the disease course, long before the emergence of traditional cardiovascular risk factors.

Variation in the axis of early dysfunction is evident across pediatric studies. Some cohorts show preserved GLS but abnormalities in circumferential strain and rotational mechanics, as demonstrated in adolescents assessed with three‐ and four‐dimensional speckle‐tracking echocardiography [21]. These rotational changes occur in the context of normal ejection fraction and tissue doppler indices, indicating that mid‐wall and subepicardial fiber behavior can be altered even when longitudinal fibers remain functionally intact. Such variation reflects differences in microvascular reserve, myocardial energetics, and developmental stage, but remains anchored in the same metabolic–microvascular pathophysiology [21].

The biological plasticity of early disease is highlighted by interventional data. In a randomized trial of 60 children and adolescents, *Nigella sativa* supplementation improved GLS and diastolic indices and reduced markers of oxidative stress, inflammation, and endothelial dysfunction [22]. These findings suggest that at least some early deformation abnormalities may be influenced by modifiable metabolic and inflammatory factors, although improvement in GLS cannot by itself establish reversal of myocardial injury. This contrasts with adult cohorts, where deformation abnormalities are more tightly linked to microvascular rarefaction and interstitial fibrosis.

In young adults, the same longitudinal‐first, diastolic‐early pattern persists. A study of asymptomatic adults with T1D showed impaired GLS and systolic strain rate despite preserved ejection fraction and normal chamber geometry, accompanied by diastolic abnormalities on both conventional and tissue doppler imaging [23]. Early‐diastolic strain rate remained normal, consistent with energetic impairment and subendocardial fiber vulnerability preceding overt diastolic recoil abnormalities.

Larger adult cohorts deepen this mechanistic picture. In the Thousand & 1 Study, arterial stiffness showed a strong independent association with impaired GLS and with diastolic function, while ejection fraction remained unrelated [24]. This coupling between vascular stiffness and deformation indices underscores the shared microvascular substrate of diabetic angiopathy and diabetic cardiomyopathy. Additional evidence from a cohort of 102 asymptomatic adults demonstrated that impaired GLS and diastolic dysfunction were common even in the absence of overt coronary disease, and that visceral adiposity was the strongest predictor of GLS impairment [25]. These findings highlight the combined influence of metabolic load, adipose‐driven inflammation, and microvascular dysfunction on subendocardial fiber performance.

Not all adult cohorts show GLS impairment, but these exceptions are informative rather than contradictory. In a study of 40 asymptomatic, normotensive adults with relatively low microvascular burden, Weber et al. found preserved GLS but clear diastolic abnormalities [26]. This pattern aligns with a diastolic‐first stage of disease, where impaired relaxation and early filling abnormalities precede measurable reductions in longitudinal systolic strain. Such findings reinforce the staged nature of T1D cardiomyopathy and highlight the influence of metabolic exposure, vascular status, and glycemic variability on the timing of mechanical abnormalities.

Acute metabolic modulation adds an additional layer of complexity. In a randomized crossover trial of 24 adults with T1D, Andreasen et al. demonstrated that acute hypoglycemia significantly increased ejection fraction, GLS, and strain rate, with these effects persisting during recovery and amplified by rebound hyperglycemia [27]. These findings show that GLS is highly sensitive to acute metabolic and catecholaminergic shifts, and that transient improvements in deformation indices may reflect stress‐induced hypercontractility rather than true structural recovery. This helps explain why some T1D cohorts exhibit normal or even supranormal GLS despite underlying microvascular or metabolic injury.

Recent interventional evidence suggests that some vascular and myocardial abnormalities in T1D may be modifiable. In patients with poorly controlled T1D, hybrid closed‐loop system (HCLS) therapy produced the greatest improvement in time in range compared with adjunctive sodium‐glucose cotransporter‐2 inhibitor (SGLT2i) therapy or intensified multiple daily insulin injections. At 12 months, HCLS and SGLT2i therapy improved GLS by 6.7% and 4.0%, respectively, compared with 2.2% with intensified multiple daily insulin therapy, alongside parallel improvements in coronary flow reserve and flow‐mediated dilatation.

Improvements in GLS and vascular indices were also associated with changes in time in range, supporting a potential link between improved glycemic control, vascular function, and myocardial deformation. As these myocardial findings are currently available as congress data, confirmation in peer‐reviewed longitudinal studies remains important [28].

Taken together, these data outline a trajectory in which early metabolic stress, oxidative injury, endothelial dysfunction, and microvascular compromise produce subtle deformation abnormalities that evolve into more fixed systolic and diastolic dysfunction as fibrosis and capillary rarefaction accumulate. GLS is therefore a sensitive marker of early systolic involvement in T1D. Emerging interventional evidence suggests that at least some deformation abnormalities may be modifiable alongside improvements in glycemic and vascular control, although the role of GLS as a longitudinal treatment‐response biomarker remains less established than in T2D. Acute metabolic and hemodynamic influences should also be considered, as they may alter GLS independently of structural myocardial change [27, 29].

The current evidence base also has important limitations. Most studies in T1D are cross‐sectional, involve relatively small cohorts, and evaluate surrogate imaging endpoints rather than long‐term cardiovascular outcomes. Consequently, although impaired GLS appears to reflect cumulative glucotoxic and microvascular injury, it remains uncertain whether longitudinal changes in GLS predict progression to overt diabetic cardiomyopathy or whether earlier intervention can meaningfully alter this trajectory. Prospective studies combining serial strain imaging with myocardial tissue characterization are needed to establish the prognostic significance of these deformation abnormalities.

## 4 Mechanistic Substrate of Myocardial Injury in Type 2 Diabetes

T2D shares several myocardial injury pathways with T1D, but insulin resistance, adiposity, altered substrate utilization, lipotoxicity and mitochondrial dysfunction may contribute more prominently to its myocardial phenotype [30]. These mechanisms create a myocardial environment characterized by energetic inefficiency, increased oxygen demand, and impaired relaxation. These potentially modifiable metabolic abnormalities may contribute to the improvements in myocardial deformation reported following cardiometabolic therapy in T2D. Comparable treatment‐response data remain much more limited in T1D [31].

Insulin resistance is central to the T2D myocardial phenotype. In the setting of insulin resistance, myocardial substrate utilization shifts towards greater reliance on fatty‐acid oxidation [32]. This shift is metabolically expensive: fatty‐acid oxidation requires more oxygen per unit of ATP generated, increasing myocardial oxygen demand [33]. Hussain et al. similarly emphasize that impaired insulin signaling in T2D reduces myocardial glucose utilization and increases reliance on fatty‐acid metabolism, contributing to reduced energetic efficiency and impaired contractile performance [13]. Over time, this metabolic inflexibility leads to mitochondrial overload, accumulation of toxic lipid intermediates, and increased oxidative stress [34].

Lipotoxicity is another defining feature. Excess circulating fatty acids enter cardiomyocytes and are stored as ceramides and diacylglycerols [35]. These intermediates activate pro‐fibrotic pathways such as TGF‐β and increase reactive oxygen species production [36]. The resulting abnormalities in myocardial energetics, relaxation and stiffness may initially contain a greater functional component, although structural remodeling and fibrosis also occur in T2D [37]. This distinction underpins the different behavior of GLS in T2D compared with T1D.

Low‐grade inflammation further contributes to myocardial dysfunction. Adipose tissue in T2D acts as an endocrine organ, releasing cytokines such as TNF‐α and IL‐6. These cytokines promote endothelial dysfunction, impair nitric‐oxide signaling, and reduce coronary microvascular reserve [38]. This inflammatory burden is amplified in individuals with obesity, hypertension, or metabolic syndrome, all of which are common in T2D [39].

## 5 Myocardial Deformation Pattern in Type 2 Diabetes

Across multiple cohorts of asymptomatic individuals with preserved ejection fraction, GLS is consistently the earliest and most sensitive marker of systolic dysfunction in T2D. Nakai and colleagues reported abnormal GLS in 43% of T2D patients with normal EF, while Tadic and Karagöz et al. independently showed strong relationships between GLS, HbA1c, and metabolic burden [40, 41, 42]. Larger datasets reinforce this metabolic gradient: in a cohort of 292 adults, Gao et al. demonstrated a clear, graded deterioration in GLS with rising HbA1c, independent of left‐atrial function, confirming that longitudinal fiber performance tracks cumulative glycemic exposure [43].

Metabolic load extends beyond glucose. Visceral adiposity is a dominant upstream determinant of myocardial vulnerability. In a 2025 cohort of 195 adults with T2D, Martinez‐Dominguez et al. showed that visceral adipose tissue mediated more than 60% of the relationship between insulin resistance and GLS, positioning visceral fat as a central driver of subendocardial dysfunction [44]. Complementary cardiac MRI data demonstrate that epicardial adipose tissue (EAT) exerts similar effects: Zhu et al. found that higher EAT volumes were independently associated with impaired LV and RV longitudinal strain and strain rate, integrating metabolic, inflammatory, and mechanical pathways into a unified adiposity‐driven mechanism [45].

Early myocardial dysfunction in T2D also reflects autonomic dysregulation. Silverii et al. showed that GLS impairment correlated with HbA1c, BMI, waist circumference, and autonomic indices, while remaining unrelated to classical microvascular complications such as retinopathy or microalbuminuria [46]. In multivariable analysis, HbA1c was the only independent determinant of GLS in this cohort, supporting an important association between glycemic burden and early systolic dysfunction. However, these findings do not exclude contributions from microvascular disease or other metabolic comorbidities [46].

Evidence for treatment‐associated improvement in GLS is currently more extensive in T2D than in T1D, and includes both SGLT2 inhibition and GLP‐1 receptor agonist‐based therapy. In a real‐world cohort of 35 well‐controlled T2D patients treated with empagliflozin or dapagliflozin, Russo et al. documented significant improvements in GLS, myocardial work efficiency, and ejection fraction over six months, accompanied by a marked reduction in global wasted work [47]. Larger observational data confirm this responsiveness across the disease spectrum: Wang et al. demonstrated that SGLT2 inhibitor therapy improved GLS across all disease‐duration strata, with the greatest benefit in early‐stage disease [48]. Importantly, both SGLT2 inhibitor use and change in EAT thickness independently predicted GLS improvement, linking metabolic therapy, adipose‐tissue modulation, and myocardial recovery.

Treatment‐associated improvements in myocardial deformation may also reflect favorable changes in vascular and metabolic function. GLP‐1 receptor agonists (GLP‐1RA) and SGLT2 inhibitors have been associated with reduced oxidative stress and improved endothelial glycocalyx integrity and arterial function in T2D [49]. These changes have occurred alongside improvements in GLS, myocardial work and NT‐proBNP, with combined GLP‐1RA/SGLT2 inhibitor therapy generally producing greater effects than either treatment alone [50]. Importantly, some vascular and myocardial benefits have been observed despite similar reductions in HbA1c, suggesting mechanisms beyond glycemic lowering alone [51]. More recently, improvement in GLS after six months of combined liraglutide and empagliflozin therapy independently predicted a lower risk of cardiovascular events over six years, supporting a potential prognostic role for serial GLS assessment [52].

Randomized evidence now extends these findings to primary prevention. In the ELUCIDATE trial, asymptomatic adults with T2D and preserved EF randomized to dapagliflozin showed significant reductions in LV dimensions, end‐systolic volume, and LV mass index, together with measurable improvements in GLS and both systolic and early‐diastolic strain rates [53]. These changes occurred without corresponding changes in NT‐proBNP or measures of insulin resistance, suggesting that deformation indices may detect treatment‐associated changes not captured by these conventional markers.

More recent evidence suggests that treatment‐associated improvements in myocardial deformation may also parallel changes in neurohumoral, oxidative and mitochondrial pathways. In patients with T2D and high or very high cardiovascular risk, treatment with a glucagon‐like peptide‐1 receptor agonist (GLP‐1RA), an SGLT2 inhibitor, or their combination improved GLS, left atrial reservoir strain and global work index compared with insulin therapy. Reductions in NT‐proBNP were associated with improvements in GLS, left atrial strain and global work index, while changes in growth differentiation factor‐15 (GDF‐15) and the mitochondrial‐derived peptide (MOTS‐c) were related to improved antioxidant capacity and myocardial mechanics. Combination therapy produced the greatest reduction in neurohumoral markers, whereas SGLT2 inhibition appeared to have a stronger effect on mitochondrial activation. These findings support a link between cardiometabolic therapy, myocardial deformation and broader neurohumoral and mitochondrial adaptation [54].

In more advanced stages of T2D cardiomyopathy, once systolic dysfunction is established, structural remodeling rather than deformation change becomes the dominant therapeutic target. In the SUGAR‐DM‐HF randomized trial, empagliflozin reduced LV end‐systolic and end‐diastolic volumes in patients with T2D or prediabetes and established HFrEF, despite no change in GLS on cardiac MRI [55]. This pattern complements the GLS improvements observed in earlier‐stage T2D, indicating that metabolic therapies improve deformation in preserved‐EF disease, whereas volumetric reverse remodeling becomes more prominent once systolic dysfunction is present.

Circumferential and radial mechanics show greater variability in T2D than in T1D. Some cohorts report preserved circumferential strain, while others identify early apical impairment [40, 41, 56]. Radial strain may be preserved or mildly increased early due to compensatory thickening but declines as fibrosis becomes more diffuse [1]. This heterogeneity reflects the influence of comorbid hypertension, obesity, and metabolic syndrome, which modulate the pattern and severity of myocardial involvement.

Markers of mechanical timing provide additional insight. Peak strain dispersion (PSD) is particularly sensitive in T2D: Li et al. showed that PSD was significantly elevated even when GLS remained normal, and strongly correlated with HbA1c, indicating that electromechanical dyssynchrony may precede overt systolic impairment [7]. Layer‐specific strain (LSS) often shows multi‐layer involvement, particularly in individuals with coexisting hypertension, reflecting a more transmural pattern of dysfunction than that typically observed in T1D [57].

Taken together, the mechanistic and imaging evidence supports a coherent model of T2D cardiomyopathy. Despite these encouraging findings, important uncertainties remain. Although multiple studies report improvements in GLS following SGLT2 inhibitor therapy, it remains unclear whether these changes represent genuine reversal of myocardial injury or reflect favorable alterations in preload, afterload, myocardial energetics, or ventricular loading conditions. Current evidence therefore supports GLS as a sensitive marker of therapeutic response, but its ability to distinguish functional recovery from structural myocardial repair remains unresolved. Future longitudinal studies integrating strain imaging with cardiac magnetic resonance tissue characterization and circulating biomarkers of fibrosis will be essential to clarify the biological significance of GLS improvement.

## 6 Future Directions

Despite increasing use of GLS for detecting subclinical diabetic cardiomyopathy, future research should move beyond determining whether myocardial deformation is abnormal and instead focus on understanding what that abnormality biologically represents. Current evidence suggests that similar reductions in GLS may occur within different pathophysiological contexts in T1D and T2D, although substantial mechanistic overlap exists.

One important priority will be the development of mechanism‐based imaging frameworks capable of distinguishing metabolically mediated myocardial dysfunction from irreversible structural injury. Rather than applying uniform GLS thresholds across all patients with diabetes, future studies should determine whether deformation patterns can be interpreted in the context of disease subtype, myocardial fibrosis, metabolic flexibility, and therapeutic responsiveness.

Similarly, longitudinal investigations should determine whether improvement in GLS reflects genuine reversal of myocardial injury or merely transient changes in ventricular loading conditions and myocardial energetics. This distinction is particularly relevant in T2D, where strain frequently improves following SGLT2 inhibitor therapy, whereas abnormalities in T1D appear more closely associated with cumulative structural remodeling. Establishing the biological meaning of longitudinal changes in myocardial deformation may ultimately prove more important than improving measurement precision alone.

Finally, future clinical trials should consider diabetic cardiomyopathy as a heterogeneous disease rather than a single pathological entity. Stratifying participants according to diabetes subtype, metabolic phenotype, or predominant mechanism of myocardial injury may reveal differences in prognosis and treatment response that remain obscured when both conditions are analyzed together. Such an approach would move strain imaging from an early detection tool towards a biologically informed biomarker capable of guiding precision cardiovascular care.

## 7 Conclusion

The evidence suggests that GLS should not be interpreted as a universal biomarker across diabetes. Rather, its biological meaning depends on disease subtype.

Although substantial mechanistic overlap exists, impaired GLS in T1D may occur in the context of cumulative glucotoxic, microvascular and fibrotic injury, whereas insulin resistance, adiposity, altered myocardial energetics and loading conditions may contribute more prominently in T2D. Importantly, GLS alone cannot distinguish these mechanisms, and its interpretation should therefore incorporate diabetes subtype, clinical phenotype and complementary imaging findings.

## Funding

The authors received no financial support for the research, authorship, and/or publication of this article.

## Ethics Statement

Not applicable.

## Consent to Participate

Not applicable.

## Consent for Publication

Not applicable.

## Conflicts of Interest

The authors declared no potential conflicts of interest with respect to the research, authorship, and/or publication of this article.

## Data Availability

No new data were generated or analyzed in this review. Data sharing is therefore not applicable to this article.
